# Multi-omics & pathway analysis identify potential roles for tumor N-acetyl aspartate accumulation in murine models of castration-resistant prostate cancer

**DOI:** 10.1016/j.isci.2022.104056

**Published:** 2022-03-11

**Authors:** Mark J. Salji, Arnaud Blomme, J. Henry M. Däbritz, Peter Repiscak, Sergio Lilla, Rachana Patel, David Sumpton, Niels J.F. van den Broek, Ronan Daly, Sara Zanivan, Hing Y. Leung

**Affiliations:** 1 Institute of Cancer Sciences, College of Medical, Veterinary and Life Sciences, University of Glasgow, Bearsden, Glasgow G61 1QH, UK; 2 CRUK Beatson Institute, Bearsden, Glasgow G61 1BD, UK

**Keywords:** Cell biology, Proteomics, Metabolomics

## Abstract

Castration-resistant prostate cancer (CRPC) is incurable and remains a significant worldwide challenge (Oakes and Papa, 2015). Matched untargeted multi-level omic datasets may reveal biological changes driving CRPC, identifying novel biomarkers and/or therapeutic targets. Untargeted RNA sequencing, proteomics, and metabolomics were performed on xenografts derived from three independent sets of hormone naive and matched CRPC human cell line models of local, lymph node, and bone metastasis grown as murine orthografts. Collectively, we tested the feasibility of muti-omics analysis on models of CRPC in revealing pathways of interest for future validation investigation. Untargeted metabolomics revealed NAA and NAAG commonly accumulating in CRPC across three independent models and proteomics showed upregulation of related enzymes, namely N-acetylated alpha-linked acidic dipeptidases (FOLH1/NAALADL2). Based on pathway analysis integrating multiple omic levels, we hypothesize that increased NAA in CRPC may be due to upregulation of NAAG hydrolysis via NAALADLases providing a pool of acetyl Co-A for upregulated sphingolipid metabolism and a pool of glutamate and aspartate for nucleotide synthesis during tumor growth.

## Highlights

• Castration-resistant prostate cancer (CRPC) remains a major clinical challenge
• Human prostate cancer cells grown as orthografts are useful pre-clinical CRPC models
• Multi-omics analysis of hormone-responsive and resistant orthografts were performed
• Altered tumoral N-acetyl aspartate and related pathway are implicated in CRPC

## Graphical abstract

**Figure:**
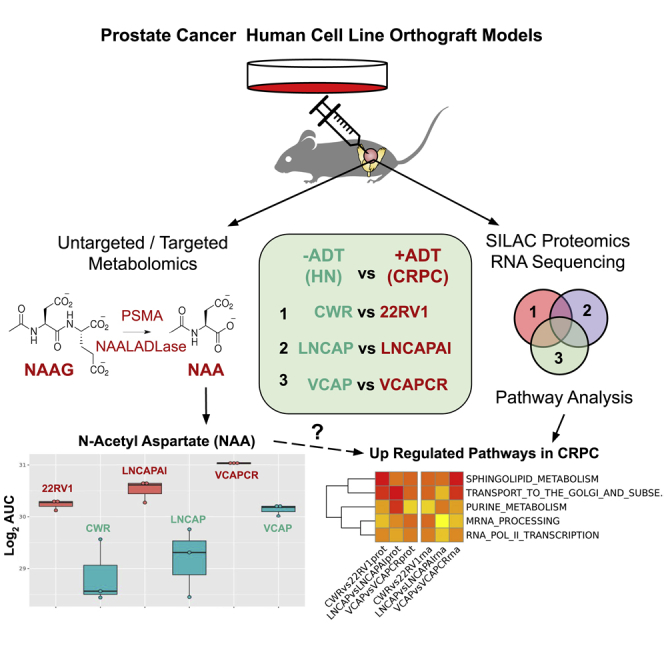

## Introduction

Prostate cancer (PC) is the most prevalent malignancy among adult men in the developed world, and PC-associated mortality continues to rise (Oakes and Papa, 2015; Smittenaar et al., 2016). Androgen deprivation therapy (ADT) remains the cornerstone of treatment of advanced prostate cancer but men on ADT will eventually develop CRPC.

Metabolic alterations have previously been studied in prostate cancer progression (Geng et al., 2018; Komatsu et al., 2012; Meller et al., 2016; Sharifi, 2013; Sreekumar et al., 2009), including CRPC (Geng et al., 2018). Sarcosine has been implicated in PC progression, with androgen receptor (AR) and the *TMPRSS-ERG* fusion gene product regulating components of the sarcosine pathway (Sreekumar et al., 2009). Lipid and cholesterol metabolism have also been shown to play a key role in prostate cancer progression and development of CRPC (Komatsu et al., 2012; Patel et al., 2018; Taylor et al., 2015).

Analysis of orthotopic xenografts derived from human PC cell models of local disease (CWR vs 22RV1) (Sramkoski et al., 1999), lymph node metastasis (LNCAP and LNCAPAI) (Horoszewicz et al., 1980), and bone metastasis (VCAP and VCAPCR) (Korenchuk et al., 2001) may identify common metabolites and pathways in CRPC across major disease stages. The acquisition of matched global untargeted omic datasets in combination at different omics levels can be a powerful tool to elucidate not only what is changed but also what is biologically important in CRPC to better inform future therapeutics (Stagljar, 2016).

## Results

### Metabolomics identifies increased N-acetyl aspartate (NAA) and N-Acetyl aspartyl glutamate (NAAG) in all three models of CRPC

**Figure 1:**
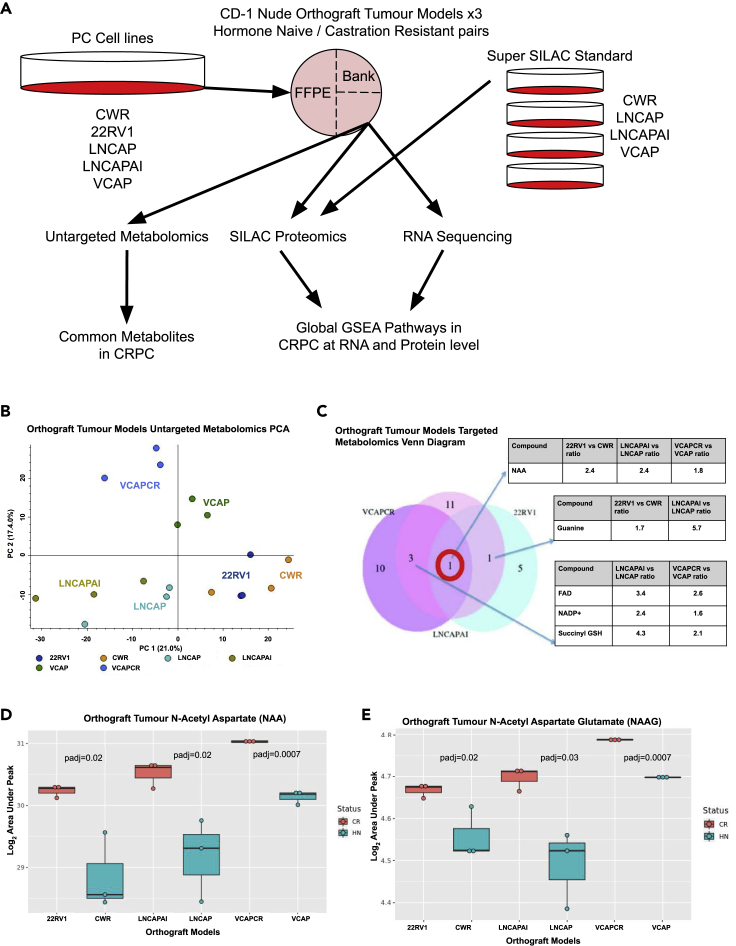
Workflow for matched metabolomics, proteomics, and RNA sequencing of three human orthograft models of CRPC with untargeted and targeted metabolomics of CRPC highlighting N-acetyl aspartate (NAA) and N-acetyl aspartate glutamate increased in CRPC tumor models (A) Three pairs of models were used each with a matched hormone naive (HN) and castration-resistant prostate cancer (CRPC) counterpart representing progressive stages of tumor from their cell line origin. CWR developed from TURP specimens representing primary disease, LNCAP developed from lymph node metastasis, and VCAP developed from vertebral bone metastasis. A quarter of each orthograft was cryo-ground and the same sample used for metabolomics, proteomics, and RNA sequencing in order to address intratumoural heterogeneity. (B) Untargeted metabolomics (n = 1,094 compounds negative ion mode) principal component analysis (PCA) plot of tumor models of CRPC using Compound Discover (Thermo). Biological tumor triplicates are shown as separate points and separation of the different tumor models can be seen. VCAPCR model tumors show the best separation between the CRPC and HN models based on their untargeted metabolite profile using the unsupervised PCA. (C) Targeted metabolomics (n = 112 compounds) Venn diagram showing NAA identified as significantly increased in all three CRPC tumors compared to the HN counterpart, fold change (FC) of area under curve (AUC) is shown for metabolites significantly increased using Welch’s t test p ≤ 0.05 in more than one model. All targeted metabolites AUC, mean, SD, FC, Welch’s t test p value, and Benjamini Hochberge (BH) adjusted p value (padj) are shown in Data S1. (D and E) Abundance of NAA (D) and NAAG (E) in HN (CWR, LNCAP, VCAP - blue/green) and CRPC (22RV1, LNCAPAI, VCAPCR - red) tumor models. Both NAA and NAAG levels are significantly increased in all three tumor models (Welch’s t test Benjamini Hochberge (BH) adjusted p value (padj). All targeted metabolites AUC, mean, SD, FC, Welch’s t test p value, and Benjamini Hochberge (BH) adjusted p value (padj) are shown in Data S1. Box and whisker plots midline represents the median Log_2_ area under curve (AUC) with hinges representing the first and third quartiles and whiskers extending to 1.5× the IQR with all data points including outliers shown.

Untargeted metabolomics was performed on the matched hormone naive (HN) and CRPC orthografts to identify commonly altered small molecule metabolites in CRPC in an unbiased manner. A principal component analysis (PCA) was performed using Compound Discoverer 3.0 (thermofisher) (CD) based on negative ion mode analysis. Figure 1B shows the PCA plot with replicates from orthograft models clustering together. The VCAP and VCAPCR model showed the greatest separation between HN and CR tumors using this unsupervised analysis. An orthogonal projection to latent structures (OPLS) discriminant analysis (DA) model was generated using Progenesis QI (Water’s) software, with prior segregation of samples into either HN or CRPC groups again based on negative ion mode analysis. The OPLS-DA loading S-plot and OPLS-DA scatterplot (Figures S1A and S1B) show replicates from individual orthografts clustering together and clear separation between HN and CR orthografts. The abundance of individual metabolites as discriminators of CR and HN tumors was then investigated. N-acetyl aspartate (NAA) represented the most increased metabolite among all three CRPC models, seen as the metabolite at top right of the S-plot (Figure S1A). Untargeted metabolomics data were then further analyzed using CD and MS2 spectrum matches for NAA and N-acetyl aspartate glutamate (NAAG) were confirmed with high confidence (Figures S1C and S1D, detailed data files are available via Mendely data portal).

Targeted metabolomics analysis of 112 compounds (Data S1, with detailed data files are available via Mendely data portal) validated the observed elevated tumoral NAA and NAAG levels in CRPC along with the nucleobase guanine increased in 22RV1 and LNCAPAI and FAD, NADP+ and succinyl GSH increased in LNCAPAI and VCAPCR (Figure 1C). Levels of tumoral NAA and NAAG were upregulated in all three CRPC orthografts (Figures 1D and 1E) (NAA - 22RV1/CWR, FC = 2.4, padj = 0.02; LNCAP/LNCAPAI, FC = 2.4, padj = 0.02; VCAP/VCAPCR, FC = 1.8, padj = 0.0007; NAAG - 22RV1/CWR, FC = 3.0, p = 0.02; LNCAP/LNCAPAI, FC = 8.7, p = 0.03; VCAP/VCAPCR, FC = 3.2, p = 0.0007; Welch’s T-test Benjamini Hochberge (BH) adjusted p value (padj).

As VCAP and VCAPCR models showed the highest levels of NAA and NAAG (Figures 1D and 1E), the VCAP model was used to test whether the increase in tumoral NAA/NAAG level was associated with corresponding changes in serum NAA/NAAG level. Targeted metabolite analysis for serum NAA and NAAG levels showed comparable levels across all experimental groups of HNPC and CRPC (Figures S1D and S1E). Therefore, enhanced accumulation of NAA/NAAG in CRPC is more likely to be a local tumor phenomenon than a systemic increase due to castration.

### Analysis of gene expression at the proteome and transcript levels

**Figure 2:**
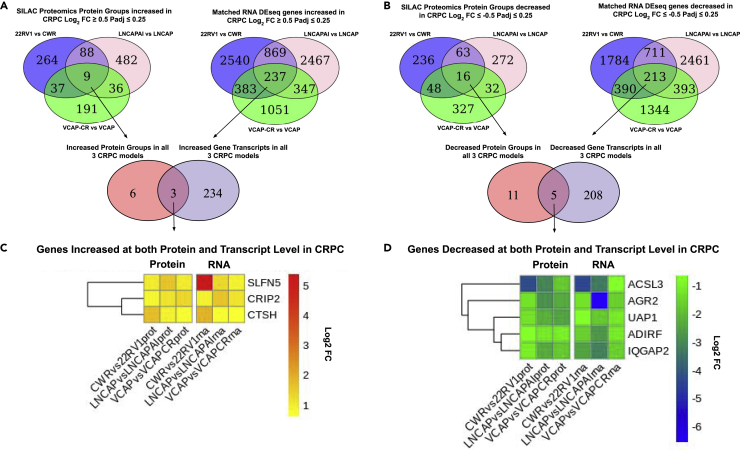
Combined SILAC proteomics and RNA sequencing overlap highlights individual increasing or decreasing genes at protein and transcript level with agreement in all three models of CRPC (A and B) Venn diagrams showing individual gene overlap at both protein (left) and transcript (right) level in three tumor models and upregulated (A) or downregulated (B) in CRPC by at least 0.5 Log_2_ Fold Change (corresponding to 1.4× fold change or 40% increase/decrease in protein abundance or mRNA reads) and P adjusted value ≤ 0.25. Protein groups were quantified by Log_2_ SILAC ratio change in CRPC and RNA by Log_2_ Fold Change normalized reads (DESeq2). Positive values show the relative increase in the CRPC tumor compared to its HN counterpart (n = 4470 total protein groups ≥1 unique peptide identified in at least two of three biological replicates). (C) SLFN5 (Log_2_ FC at protein level - CWRvs22RV1 1.04, (padj = 0.2), LNCAPvsLNCAPAI, 1.58 (padj = 0.01), VCAPvsVCAPCR, 1.05 (padj = 0.006). Log_2_ FC at RNA level CWRvs22RV1, 5.37 (padj = 7.1 × 10^−36^), LNCAPvsLNCAPAI, 1.32 (padj = 6.7 × 10^−31^), VCAPvsVCAPCR, 0.93 (padj = 3.3 × 10^−13^), CRIP2 (Log_2_ FC at protein level - CWRvs22RV1 0.88, (padj = 0.2), LNCAPvsLNCAPAI, 1.16 (padj = 0.01), VCAPvsVCAPCR, 1.33 (padj = 0.006). Log_2_ FC at RNA level CWRvs22RV1, 0.96 (padj = 7.1 × 10^−36^), LNCAPvsLNCAPAI, 1.63 (padj = 6.7 × 10^−31^), VCAPvsVCAPCR, 1.11 (padj = 3.3 × 10^−13^) and CTSH (Log_2_ FC at protein level - CWRvs22RV1 1.73, (padj = 0.2), LNCAPvsLNCAPAI, 0.90 (padj = 0.01), VCAPvsVCAPCR, 0.71 (padj = 0.006). Log_2_ FC at RNA level CWRvs22RV1, 1.89 (padj = 7.1 × 10^−36^), LNCAPvsLNCAPAI, 0.73 (padj = 6.7 × 10^−31^), VCAPvsVCAPCR, 0.61 (padj = 3.3 × 10^−13^) are the only three genes with increased expression in all three models by both protein and mRNA quantification, above set cutoff levels, with Log_2_ FC increase at protein and RNA level shown increasing from yellow to red in the heatmap. SLFN5 shows marked increase at both protein and RNA level and has been subsequently further investigated (Martinez et al., 2020). (D) ACSL3, AGR2, UAP1, ADIRF, and IQGAP2 were the only five genes with decreased expression in all three models by both protein and mRNA quantification, below set cutoff levels. Log_2_ Fold Change decrease at protein and RNA level in CRPC is shown as negative values decreasing from green to blue in the heatmap. These genes encode proteins with diverse functions from fatty acid synthesis to protein folding, protein metabolism, and adipogenesis and cell adhesion.

Individual genes commonly up- or downregulated at both the proteome and transcriptome level may suggest steady state changes for the implicated genes in CRPC (Liu et al., 2016). In order to map the global proteome and transcriptome changes onto downstream metabolite changes in CRPC, both quantitative Stable Isotope Labeling with Amino acids in Cell culture (SILAC)-based proteomics and RNA sequencing were analyzed on the matched HN and CRPC tumors in parallel. Analysis of gene expression at the proteome and transcript levels in the three HN (CWR, LNCAP, and VCAP) and isogenic CRPC models (22RV1, LNCAPAI, and VCAPCR) is shown in Figures 2A and 2B (panel A showing upregulated genes and panel B showing downregulated genes). 1,107 proteins and 7,894 transcripts were upregulated (Log_2_FC ≥ 0.5, Padj≤0.25) and 994 proteins and 7,296 transcripts downregulated (Log_2_FC ≤ −0.5, Padj≤0.25) among all models. Comparing conserved upregulated proteins (n = 9) and transcripts (n = 237) in all three models, three genes were upregulated in CRPC at both protein and transcript levels (Figure 2C), namely Human Schlafen 5 (SLFN5), Cysteine-Rich Protein 2 (CRIP2), and Cathepsin H (CTSH). Genes downregulated at both protein and transcript levels in all three CRPC models (Figure 2D) were ACSL3, AGR2, UAP1, ADIRF, and IQGAP2, encoding proteins with diverse functions involving fatty acid synthesis, protein folding, and adipogenesis. As only a few genes were consistently altered at both expression levels, an alternative multi-omic pathway analysis was performed with overlap at the pathway level in an attempt to address redundancy between individual gene expression levels.

### Gene set enrichment analysis (GSEA) of Reactome Pathways of the CRPC proteome and transcriptome highlights regulated pathways in CRPC at both omic levels

**Figure 3:**
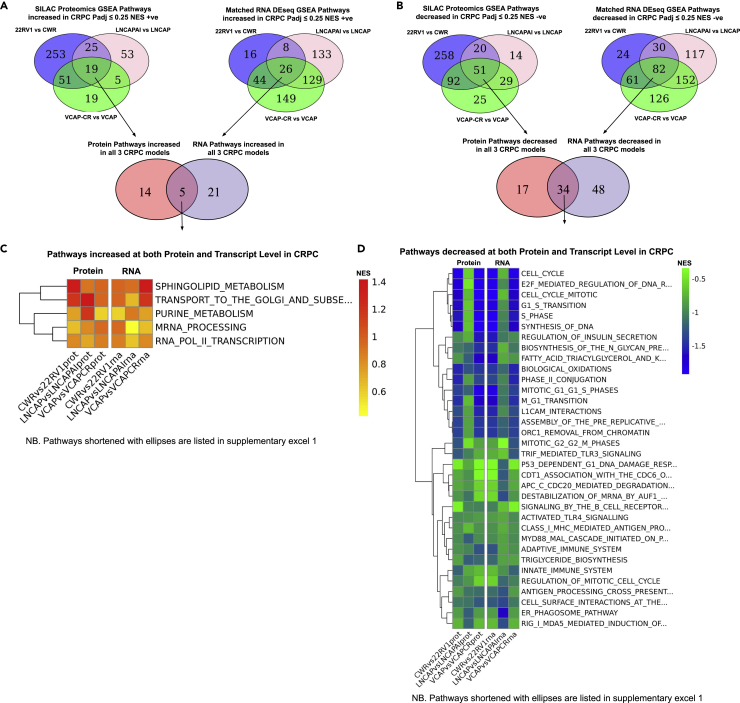
Combined gene set enrichment analysis (GSEA) of Reactome Pathways at the protein and RNA level highlights pathways increased or decreased with agreement in all three models of CRPC (A and B) Venn diagrams showing overlap of Reactome Pathways increased in CRPC (A) or decreased in CRPC (B) at both protein (left) and transcript level (right) in the three tumor models. Protein groups were ranked by Log_2_ SILAC ratio change for GSEA enrichment analysis. Normalized enrichment score (NES) was either positive (increased) (A) or negative (decreased) (B) in CRPC with P adjusted value ≤ 0.25 (BH) for pathway enrichment. (C) Heatmap of NES shows five pathways increased (positive NES) (C) at both protein and transcript level in all three CRPC tumor models: Sphingolipid Metabolism, Transport to the Golgi and subsequent modification [of proteins], Purine metabolism, mRNA processing, and RNA POL II Transcription. Unbiased clustering by rows of pathways by Euclidean distance is shown by the tree diagram (left) of heatmap. Full titles of pathways with ellipses are listed in Data S2. (D) Heatmap of NES of pathways decreased (negative NES) (D) at both protein and transcript level in all three CRPC tumor models shows 34 decreased pathways showing cell cycle and immune pathways predominantly. Full titles of pathways with ellipses are listed in Data S2.

To identify biologically important pathways dysregulated in CRPC, overlap analysis was performed between the three tumor models of positively enriched (upregulated) or negatively enriched (downregulated) pathways by normalized enrichment score (NES) at both protein and transcript levels (Figures 3A and 3B, with panel A showing pathways positively enriched and panel B showing pathways negatively enriched). Commonly up/downregulated pathways at the protein and transcript levels across the three tumor models of CRPC are shown as heatmaps of NES for each Reactome pathway. Only five pathways were found to be increased (Figure 3C), while 34 pathways were decreased in CRPC (Figure 3D; Data S2). Unbiased hierarchical clustering of pathways by Euclidean distance across the three tumor models is shown by the tree diagram to the left of the pathway heatmaps (pathway names with ellipses are listed in full in Data S2). Collectively, pathway analysis with agreement at both protein and RNA level showed increases in two metabolic pathways: (1) Sphingolipid metabolism which clusters with “Transport to the Golgi and Subsequent Modification”, and (2) Purine Metabolism which clusters with mRNA processing and RNA Pol II transcription (Figure 3C). The Reactome pathway for aspartate/asparagine metabolism (containing the metabolites NAA/NAAG) was not found to be significantly upregulated. Downregulated pathways comprised mainly of cell cycle and immune response-associated pathways and no specific downlegulated metabolic pathways were identified. Hence, it was unclear how and if the upregulated (protein and RNA) pathways observed related to upregulated metabolites of interest in CRPC (namely NAA/NAAG).

### Multi-omic level analysis of NAA/NAAG pathway genes identify upregulation of N-acetylated alpha-linked acidic dipeptidases (NAALADases)

**Figure 4:**
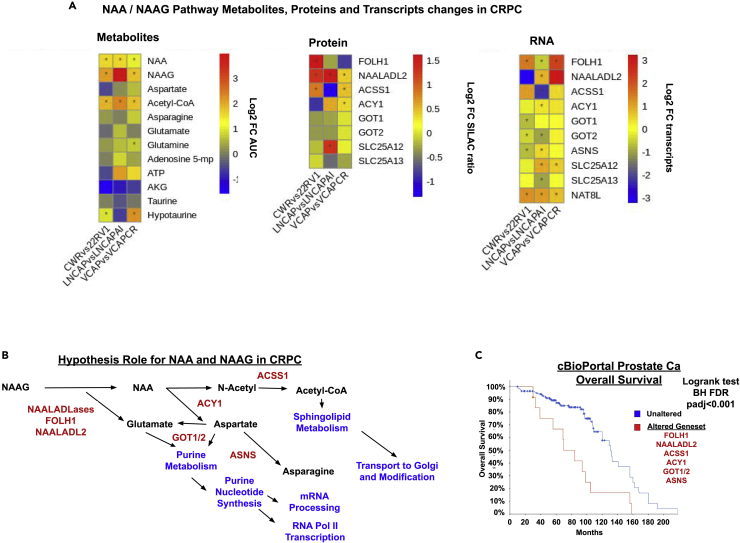
NAA/NAAG pathway metabolites, proteins, and transcripts suggest a role for NAA in the support of CRPC-enriched pathways identified (A) Heatmaps of metabolites, protein, and transcript (left to right) changes in CRPC for identified and quantified NAA/NAAG-related small molecule metabolites and genes comprising the Reactome pathway for aspartate/asparagine metabolism with the addition of ACY1 and ACSS1. Heatmap colors represent the mean Log_2_ FC in CRPC tumors (22RV1, LNCAPAI, and VCAPCR) compared to the HN counterpart tumors (CWR, LNCAP, and VCAP) (mean Log_2_ FC of CRPC model minus mean Log_2_ FC of HN model with positive value representing an increase in abundance in the CRPC model). Star indicates significance with Welch’s T-test p value ≤0.05. (B) Hypothesis flow diagram linking NAA/NAAG metabolism to enriched pathways identified in CRPC (blue) and metabolic enzymes (red). The position of NAA metabolizing enzymes (red) identified and quantified above in the heatmaps at protein and transcript level in models of CRPC are shown. (C) Survival analysis of publicly available clinical PC datasets via cBioPortal (Grasso et al., 2012; Hieronymus et al., 2014; Taylor et al., 2010) showing reduced overall survival of patients with copy number amplifications in the geneset of enzymes associated with NAA/NAAG metabolism (FOLH1, NAALADL2, ACSS1, ACY1, GOT1/2, and ASNS) (Log-Rank test BH padj = 5.99 × 10^−4^). Altered group n = 12 median overall survival = 70 months, unaltered n = 140 median overall survival = 131 months. In addition, there was also association with disease-free survival (Log-Rank test BH FDR padj = 4.03 × 10^−3^, altered n = 4 median disease free survival = 4.11 months, unaltered n = 274 median overall disease free survival = 110.33 months.

We hypothesized that increased levels of NAA/NAAG in CRPC may be associated with the observed upregulated pathways in CRPC, namely the sphingolipid and purine metabolic pathways, Transport to the Golgi and subsequent modification, and mRNA processing and RNA Pol II transcription (Figure 3C). The aspartate/asparagine Reactome metabolic pathway was interrogated at all three omic levels for changes in metabolite abundance, protein abundance, and transcript reads in CRPC (Figure 4A). We observed elevated levels of N-acetyl alpha-linked dipeptidases (NAALADLases) in CRPC when compared to the respective HN tumors, namely FOLH1 and NAALADL2 as upregulated proteins and transcripts in Figure 4A. NAALADLase (or prostate-specific membrane antigen/PSMA or FOLH1) is a clinically relevant marker for CRPC. N-acetyl alpha-linked dipeptidases (FOLH1 and NAALADL2) convert NAAG to NAA. Taken together, elevated levels of FOLH1 and NAALAD2 are consistent with increased levels of NAA and NAAG in CRPC tumors (Figures 1D and 1E).

NAALADL2 was significantly increased at the protein level in all three CRPC models while FOLH1 (or PSMA) was upregulated at the RNA level in all three CRPC models (Figure 4A). The varying patterns of FOLH1 and NAALADL2 expression at protein and RNA levels among the three sets of CRPC may reflect the overlapping nature of their enzymatic function (Whitaker et al., 2014). Of note, *NAT8L* transcripts (the synthetic enzyme of NAA) were also observed as increased in CRPC across all three models, but NAT8L was not detected at the protein level. Collectively, multi-omic analysis based on the observed metabolic alterations in CRPC demonstrated a range of cellular strategies which may explain increased levels of tumoral NAA in CRPC.

Accumulated NAA can be metabolized by aminoacylase (ACY1) releasing the N-acetyl group and aspartate. ACY1 is increased in LNCAPAI and VCAPCR at the protein level and across all three models at the transcript level (Figure 4A). The N-acetyl group can then be converted to acetyl-CoA via mitochondrial acetyl-CoA synthetase (ACSS1) required for upregulation of the sphingolipid metabolism implicated in Figure 3C. Aspartate released from NAA via ACY1 can be converted to glutamate via glutamic-oxaloacetic transaminase ½ (GOT1/2) and transported via SLC25A12/13 exchanging glutamate for aspartate across the inner mitochondrial membrane. SLC25A12/13 and GOT1/2 are slightly upregulated at both protein and transcript levels in all three CRPC models (Figure 4A). Aspartate may be further converted to asparagine via asparagine synthetase (ASNS) also increased at transcript level in 22RV1 and LNCAPAI models of CRPC in keeping with previous studies on ASNS in CRPC (Sircar et al., 2012). Collectively, our analysis supports the notion that NAA accumulation may support CRPC-associated pathways as a potential source of acetyl-CoA and aspartate (Figure 4B) (Bogner-Strauss, 2017).

The combination of genes associated with NAA/NAAG and downstream upregulated pathways were assessed for survival effects in publicly available PC datasets using cbioportal, all prostate cancer (primary and metastatic) cases with data on overall survival, gene copy number, and amplification status from multiple clinical cohorts (Grasso et al., 2012; Hieronymus et al., 2014; Taylor et al., 2010). The gene signature of FOLH1, NAALADL2, ACSS1, ACY1, GOT1/2, and ASNS showed significantly reduced overall survival (Log-Rank test BH FDR padj = 5.99 × 10^−4^, altered n = 12 median overall survival = 70 months, unaltered n = 140 median overall survival = 131 months) in keeping with increased NAA/NAAG metabolic pathways in CRPC being associated with shorter patient (overall and disease free) survival. The altered group had significantly greater proportion with ADT (Chi-Squared Test q < 10^−10^) and metastatic sites (Chi-Squared Test q = 1.17 × 10^−8^) along with higher Gleason Grade (Chi-Squared Test q < 10^−10^).

## Discussion

The objective of our study was to test the feasibility of applying a multi-omics approach on an *in vivo* panel of paired HN and CRPC tumor models to uncover novel pathways that may otherwise not be identified. The highlighted pathways can then be formally evaluated in future research. Data presented in our report will also provide an invaluable comprehensive data rich resource in prostate cancer research. This study incorporates an *in vivo* untargeted multi-omics approach to understanding the metabolomic, proteomic, and transcriptomic changes occurring in three tumor models of CRPC. N-acetyl aspartate (NAA) and N-acetyl aspartate glutamate (NAAG) were identified as significantly increased in all three models of CRPC suggesting a common feature, highlighting a potential role of these metabolites in CRPC, and the need for future research in this area. Combined proteomic and transcriptomic analysis identified increased enrichment between the three CRPC models in sphingolipid and purine metabolic pathways (Figure 3). Sphingolipid metabolism has not previously been associated with CRPC but has previously been implicated in PC metabolic dysregulation (Meller et al., 2016). Our recent lipidomics analysis also confirms increases in sphingolipid species in CRPC cell line models (Blomme et al., 2020).

In our report, we identified accumulation of NAA in CRPC. NAA has primarily been studied as a neuronal metabolite and is the most abundant small molecule metabolite in neuronal tissue (Baslow, 2002; Edden et al., 2007; Moffett et al., 2006). In neurons, NAA is synthesized by aspartate N-acetyltransferases (NATs) and primarily NAT8L which is mainly expressed in the brain (Wiame et al., 2009). NAT8L was only detected at the RNA level in our analysis but significantly increased across all CRPC models (Figure 4A). Whether NAA is synthesized in prostate tumor by NAT8L or accumulated by another mechanism remains elusive. NAA can also be produced by NAALADLases (FOLH1 and NAALADL2) through hydrolysis of NAAG releasing NAA and glutamate. NAALADL2 was significantly increased at the protein level in all three CRPC models while FOLH1 (or PSMA) was upregulated at the RNA level in all three CRPC models (Figure 4A). Collectively, our data from steady state expression analysis point to upregulated activities for the pathway involving FOLH1 and NAALADL2 in converting NAAG to NAA and glutamate. The varying patterns of FOLH1 and NAALADL2 expression at protein and RNA levels among the three sets of CRPC may reflect the overlapping nature of their enzymatic function. NAALADL2 has been shown to be upregulated in prostate cancer promoting migration and metastasis (Whitaker et al., 2014) and more recently Simpson et al. showed the genomic location of NAALADL2 (3q26.31–32 locus) as a region with amplifications or copy number gains found to be more prevalent in aggressive PC and associated with significant reduction in disease-free survival (Simpson et al., 2020). Applying our geneset associated with NAA/NAAG and downstream upregulated pathways from proteomics and RNAseq (namely FOLH1, NAALADL2, ACSS1, ACY1, GOT1/2, and ASNS) identified reduced patient (overall and disease-free) survival, along with associated features of aggressive disease with high Gleason scores, metastasis, and fraction of genome altered (Figure 4C), with patient-related parameters in analyzed clinical cohorts presented in Data S3.

FOLH1/PSMA has been widely studied in prostate cancer as a theranostic target, and its expression is suggested to increase upon ADT (Meller et al., 2015). PSMA or GCPII has also been implicated in the storage and release of glutamate through hydrolysis of NAAG in multiple cancer types (Nguyen et al., 2019). Redundancy between FOLH1 and NAALADL2 may be important from our study as primarily NAALADL2 appears to be commonly increased in CRPC but FOLH1 is markedly upregulated in the 22RV1 vs CWR primary PC model (Figure 4A). Increased glutamate released by NAAG hydrolysis via NAALADLases or exchanged for aspartate can also support pathways observed to be increased at protein and transcript level in all three CRPC models (Figure 3C), including purine metabolism, downstream purine nucleotide synthesis, and subsequent RNA Pol II transcription for mRNA synthesis.

Accumulated NAA may act as both a metabolic reservoir of acetyl Co-A and a secreted signaling metabolite promoting a pro-inflammatory tumor micro-environment conducive to CRPC (Sciarra et al., 2016). NAA has been shown to interact with adipocytes promoting a pro-inflammatory niche (Huber et al., 2019). NAA may also act on tissue-associated macrophages, increased in the periprostatic adipose tissue of CRPC tumors (Gucalp et al., 2017), increasing inflammatory cytokine release (Davies et al., 2017; Ribeiro et al., 2012).

The major limitation of this study is the use of a small number of genetically distinct tumors from orthotopic xenograft models derived from established human prostate cancer cell lines. Inclusion of additional pairs of HN and CRPC would better represent tumor heterogeneity of clinical disease. A further limitation of the model used is the lack of an adaptive immune response due to xenograft tumors. Another key limitation of this report is the lack of formal validation experiments. As the primary objective of our study was to evaluate the feasibility of a multi-omics analysis on a panel of paired HN and CRPC tumor models, we propose that future validation experiments are warranted. Collectively, our analysis supports the notion that NAA accumulation may support CRPC-associated pathways as a source of acetyl-CoA and aspartate, akin to NAA metabolism seen in the nervous system (Mehta and Namboodiri, 1995; Moffett et al., 2006). Based on our hypothesis, increased acetate and aspartate metabolism will be expected in CRPC. Data on acetate are not available from the analysis performed and will require a separate round of GC-MS analysis. Available data on aspartate from our dataset were not conclusive (see data on aspartate contained in Data S1). Nonetheless, we did observed a trend (though not reaching statistical significance) for increased aspartate in LNCaP-AI and VCAP CRPC orthografts when compared to the respective HNPC orthografts. Formal metabolic tracing experiments will be required to formally test the value of NAA/NAAG-mediated metabolism in supporting CRPC metabolism via acetyl-CoA and aspartate as intermediate metabolites.

Based on our multi-omics analysis, we hypothesize that NAA accumulation partly through the action of upregulation of NAALADLases may result in a survival advantage in CRPC by providing a source of acetate and aspartate feeding into both sphingolipid metabolism and purine metabolism, the only increased metabolic pathways at both protein and RNA level across all the three CRPC models (Figure 4A). This hypothesis may be tested in future studies employing labeled NAA lipidomics to access the fate of NAA acetate in CRPC models. Sphingolipid metabolism in particular has been previously implicated in prostate and other cancers, particularly in pro-survival and therapeutic resistance (Voelkel-Johnson et al., 2018). This highlights NAA metabolism and sphingolipid metabolism as potential common metabolic strategies for future therapeutics in CRPC.

## STAR★Methods

### Key resources table

**Table:** 

REAGENT or RESOURCE	SOURCE	IDENTIFIER
**Biological samples**		

CWR tumour ×3	this paper	N/A
22RV1 tumour ×3	this paper	N/A
LNCAP tumour ×3	this paper	N/A
LNCAPAI tumour ×3	this paper	N/A
VCAP tumour ×3	this paper	N/A
VCAPCR tumour ×3	this paper	N/A

**Chemicals, peptides, and recombinant proteins**		

RPMI 1640	Gibco	Cat#31870025
RPMI 1640 minus Phenol Red	Gibco	Cat#32404014
Glutamine 2mmol	Gibco	Cat#25030081
Charcoal Stripped Foetal Bovine Serum (CSS) 10%	Gibco	Cat#12676029

**Deposited data**		

Proteomics Raw and Analysed - Proteome Exchange/Mendeley Data	this paper	PRIDE: PXD021428 (http://www.ebi.ac.uk/pride/archive/projects/PXD021428) and Mendeley Data: https://data.mendeley.com/datasets/6jz2y44w4x/1
Metabolomics Raw and Analysed - Mendeley Data	this paper	Mendeley Data: https://data.mendeley.com/datasets/6jz2y44w4x/1
RNA sequencing - ArrayExpress / Mendeley Data	this paper	ArrayExpress: E-MTAB-9831 (https://www.ebi.ac.uk/arrayexpress/experiments/E-MTAB-9831/) and Mendeley Data: https://data.mendeley.com/datasets/6jz2y44w4x/1

**Experimental models: Cell lines**		

CWR	Case Western Reserve University, Cleveland, Ohio	N/A
22RV1	ATCC	CRL-2505
LNCAP	ATCC	CRL-1740
LNCAPAI	Newcastle University, UK	N/A
VCAP	ATCC	CRL-2876

**Experimental models: Organisms/strains**		

CD-1 Nude Mouse prostate orthograft model castrated and uncastrated	charles river	Crl:CD1-Foxn1^nu^

**Software and algorithms**		

Maxquant v. 1.5.2.8	(Tyanova et al., 2016a)	https://www.maxquant.org/
Perseus v. 1.5.2.4	(Tyanova et al., 2016b)	https://www.maxquant.org/perseus/
Progenesis	Nonlinear Dynamics	http://www.nonlinear.com/
Compound Discoverer	Thermo	https://mycompounddiscoverer.com/
Tracefinder	Thermo	https://planetorbitrap.com/tracefinder
R version 3.5.2 (2018-12-20)	CRAN	https://cran.r-project.org/
TopHat 2	(Kim et al., 2013)	https://ccb.jhu.edu/software/tophat/index.shtml
DESeq2	(Love et al., 2014)	http://www.bioconductor.org/packages/release/bioc/html/DESeq2.html

**Other**		

FASP membrane of 30 KDa pore size (15 mL Falcon)	Millipore	Cat#UFC903024
Proteomics offline high pH fractionation ×21 fractions	Dionex	Foxy Jr. FC144 fraction collector

### Resource availability

#### Lead contact

Further information and requests for resources and reagents should be directed to and will be fulfilled by the Lead Contact, Professor Hing Y Leung (h.leung@beatson.gla.ac.uk).

#### Materials availability

This study did not generate new unique reagents.

### Experimental model and subject details

#### Modeling CRPC using a pre-clinical orthotopic murine transplantation model

Orthograft tumours from hormone naïve (HN) and the respective isogenic castration resistant (CR) cell lines were obtained and processed for multiple omic level analysis as illustrated in Figure 1A and described previously (Martinez et al., 2020). CWR, LNCAP and VCAP human PC cell models were selected due to being commonly studied with CR versions maintaining AR expression and representing local, lymph node and bone metastasis stages of human PC. Triplicates were used for each HN and CR model resulting in 18 tumours studied: 9 HN control tumours (3× CWR, 3× LNCAP and 3× VCAP) and 9 CR tumours (3× 22RV1, 3× LNCAPAI and 3× VCAPCR).

#### Orthograft tumour models

Human PC cell lines were authenticated using the Promega GenePrint 10 System. CWR - RRID:CVCL_LI38, 22RV1 - RRID:CVCL_1045, LNCAP-RRID:CVCL_4783, LNCAPAI - RRID:CVCL_4791 and VCAP - RRID:CVCL_2235. Hormone naïve cell lines CWR, LNCAP and VCAP were maintained in RPMI 1640 with 2 mmol Glutamine and 10% Foetal Bovine Serum (FBS). Charcoal Stripped FBS (CSS) was used for maintenance of CR 22RV1 and LNCAPAI cell lines. 14×10^6^ PC cells in serum free RPMI were mixed with matrigel (1:1), with final volume of 50 μl, and orthotopically injected into the anterior prostate of 10 week old male CD-1 Nude mice (Charles River Labs) +/- surgical castration (Project Licence P5EE22AEE), reviewed by local ethics committee in full compliance with UK Home Office regulations (UK Animals (Scientific Procedures) Act 1986). Uptake rate for each model was CWR 100% (n = 3/3), 22RV1 83% (n = 5/6), LNCAP 15% (n = 3/20), LNCAPAI 50% (n = 3/6), VCAP 50% (n = 5/10), VCAPCR 70% (n = 7/10). The first 3 tumours from each model was used with a quarter snap-frozen and cryo ground in liquid nitrogen for protein, metabolite and RNA extraction using 4% SDS, methanol/ acetonitrile/water (5:3:2 ratio at 4°C) and RNeasy mini kit (Qiagen) respectively (Figure 1A). Whole blood was sampled at the time necropsy with approximately 1 mL obtained by inferior vena cava puncture and approximately 0.5 mL of serum obtained by centrifugation at 2000 rcf for 10 minutes at 4°C.

### Method details

#### Cell culture

Prostate Cancer (PC) cell line models CWR, 22RV1, LNCAP and LNCAPAI selected for study were tested in house for authentication and matching to known database using Promega GenePrint 10 Kit (multiplex PCR).

Hormone naïve cell lines CWR LNCAP and VCAP were maintained in RPM1 1640 with Phenol Red minus Glutamine (Gibco Cat 31870025) purchased from ThermoFisher. Glutamine was supplemented to media at 2 mmol concentration. Media was also supplemented with 10% Foetal Bovine Serum (FBS) to produce Full Media (FM) conditions. Castration Resistant cell lines LNCAPAI and 22RV1 were maintained RPM1 1640 minus Phenol Red and minus Glutamine (Gibco Cat 32404014) purchased from ThermoFisher. Phenol red free media was used to remove any effects of phenol on growth under androgen deprived conditions as phenol has been shown to have weak oestrogenic activity bearing a similar structure to non-steroidal Oestrogens (Berthois et al., 1986). Glutamine was also supplemented at 2 mmol concentration and 10% Charcoal Stripped Foetal Bovine Serum (CSS) was used to produce Charcoal Stripped (CS) androgen deprived media conditions, removing all steroid hormones and growth factors, including androgens, by the FBS charcoal filtration process.

#### LC-MS untargeted metabolomics protocol

A Q-Exactive Plus Orbitrap mass spectrometer (Thermo Scientific, Waltham, MA, USA) was used together with a Thermo Ultimate 3000 HPLC system for untargeted metabolomics. Setup of the HPLC system involved a ZIC-pHILIC column (SeQuant, 150 × 2.1 mm, 5 μm, Merck KGaA, Darmstadt, Germany), and a ZIC-pHILIC guard column (SeQuant, 20 × 2.1 mm). Initial HPLC mobile phase of 20% 20 mM ammonium carbonate was used, at pH 9.4, with 80% acetonitrile concentration. Tumour extracts (5 μL) were injected and metabolites were separated over a 30 minute mobile phase gradient. This was made by decreasing the acetonitrile concentration to 20%, using a flow rate of 200 μL/min and column temperature set at 45°C. The total analysis time for untargeted analysis including MS2 acquisition was 37 minutes per sample.

All metabolites were detected across a mass range of 75-1000 m/z using the Q-Exactive Plus mass spectrometer at a higher resolution of 70,000 (MS1) and 17.500 (MS2) with top 10 ions fragmented. Electrospray ionization was used and polarity switching was not enabled. This was because of improved quantification without polarity switching allowing time for fragmentation as well. Therefore negative and positive ion modes were performed and analysed separately. Lock masses were used and the mass accuracy obtained for all metabolites was below 5 ppm. Data were acquired with Thermo Xcalibur software.

#### Untargeted metabolomics analysis

Raw data were initially analysed using Progenesis QI (Water’s) and subsequently re-analysed using Compound Discoverer (Thermo Scientific v3.0). In Progenesis QI retention times were aligned to the pooled sample to account for any variations in retention time on column between samples during the analysis period. A total of 4,055 compounds passed the initial feature detection stage in Progenesis. Data on feature for individual compounds were processed by application of a normalisation factor calculated by comparison of the median of the log abundance ratio of all signals. This assumes that the majority of compounds do not change between samples and uses these compounds to calculate a normalisation factor for each sample which is then applied to all compounds within that sample. Orthogonal Projection to Latent Structures (OPLS) Discriminant Analysis (DA) model was generated using the Progenesis software suit, with the prior segregation of samples into either HN or CR groups. This allows the model to determine the fundamental differences between HN- and CR- PC samples and scoring each variable (metabolite) based on its ability to discriminate HN from CRPC in multidimensional space (Figure S1).

Using Compound Discoverer software (Thermo Scientific v3.0), retention times were aligned across all sample data files (maximum shift 2 min, mass tolerance 5 ppm). Unknown compound detection (minimum peak intensity 1e^6^) and grouping of compound adducts was carried out across all samples (mass tolerance 5 ppm, RT tolerance 0.5 min). Missing values were filled using the software’s Fill Gap feature (mass tolerance 5 ppm, S/N tolerance 1.5). No further normalisation factor was applied during the compound discoverer analysis. Feature identification was achieved by matching the mass and retention time of observed peaks to an in-house database generated using metabolite standards (mass tolerance 5 ppm, RT tolerance 0.5 min). Peak annotations were further confirmed using mzCloud (ddMS2) database search (precursor and fragment mass tolerance of 10 ppm, match factor threshold 50) (Figure S1B).

#### LC-MS targeted metabolomics protocol

Targeted metabolomics analysis employed the same HPLC setup as untargeted but with shorter gradient. Tumour or serum (taken at time of tumour harvesting) samples (5 μL in total injected) metabolites were separated by a 15 minute mobile phase gradient with an overall analysis time of 23 minutes per sample. Metabolites mass range was 75-1000 m/z with the mass spectrometer set at a resolution of 35,000 (at 200 m/z). Electrospray ionisation was used and also polarity switching was enabled to allow both positive and negative ion metabolites to be analysed in the same run. Lock masses was used and the mass accuracy obtained for all metabolites was below 5 ppm. Data from the Exactive Orbitrap mass spectrometer were acquired using Thermo Xcalibur software.

The area under the peak of compounds was calculated using Thermo TraceFinder software. Metabolites were identified by matching the exact mass of the singly charged ion and by also matching the known retention time of a commercial metabolite standards library on the HPLC column setup described above. Metabolite intensities were not further normalised as this was performed during the extraction; the samples were normalised by ground tumour weight or serum volume at 1:20 ratio of tumour tissue or serum to lysis buffer. The above methods are standardly used by the metabolomics service and have been previously published by Kuntz et al. (Kuntz et al., 2017).

Metabolite standards for N-acetyl-aspartate (NAA cat 00920-5G) and N-acetyl-aspartate glutamate (NAAG cat A5930-25MG) were purchased from Sigma Aldrich and were confirmed to be the metabolites identified. Later NAA and NAAG were added to the metabolite library with expected mass and retention time of 174.0403 m/z, and retention time 8.57 minutes for NAA and 303.0834 m/z, 10.10 minutes for NAAG. Data S1 shows the 112 compounds analysed by targeted metabolomics with expected retention time in minutes.

#### SILAC standard development

For SILAC standard generation, cell lines were grown using 100% dialysed serum conditions. Dialysed FBS (dFBS) was purchased from ThermoFisher to supplement full media conditions. Charcoal Stripped Serum (CSS) was dialysed in house by submerging CSS in dialysis membrane under continuous flow of water for 8 hours. Dialysed CSS (dCSS) was then filter sterilised to 0.2 micron and used to supplement SILAC charcoal stripped media.

RPMI SILAC media minus Arginine and Lysine with and without phenol red was purchased from ThermoFisher. Standard RPMI 1640 conditions were replicated by replacing Arginine and Lysine with their heavy labelled SILAC amino acid at the same concentration Arginine (Arg10) at 200 mg/L and Lysine (Lys8) at 40 mg/L. SILAC RPMI phenol free was used for SILAC CS media this was purchased from ThermoFisher only available minus Glucose which was added back to 2000 mg/L.

#### Tissue processing for *in vivo* proteomics

A quarter of frozen tumour was processed for proteomics by initial cryogrinding and mixing the whole quarter of the tumour to provide a representative sample. Approximately 20 mg of cryoground tumour was then added to a precellys homogenisation tube along with 200 μL of 4% SDS lysis buffer and processed at room temperature on the precellys machine. The lysate was then removed, boiled at 95°C for 5 minutes and sonicated prior to centrifugation.

Each of the four SILAC labelled cell lysates (CWR, LNCAP, LNCAPAI, and VCAP) were mixed at a 1:1:1:1 ratio to ensure equal contribution to the super SILAC standard. Mixed super SILAC standard was then aliquoted and stored at -80°C. Each tumour sample (18 samples representing biological triplicates of 3 tumour models of HN and matched CRPC (3 × 2 × 3 = 18)) was mixed at 1:1 ratio with the super SILAC standard prior to FASP and trypsin digestion. The amount of peptide material required for each analysis was approximately 500 μg. It was therefore required to perform FASP with approximately 1000 μg of protein (500 μg of tumour sample and 500 μg of super SILAC standard) due to expected 50% material loss during the FASP processing (Boersema et al., 2013).

Offline reverse phase high pH fractionation technique has previously been described (Wang et al., 2011) and here it is applied to a SILAC mixture to improve the number of peptide identification by reducing the complexity of the sample by splitting each sample prior to MS analysis. A C18 column (250 × 4.6 mm i.d. – Durashell RP (5 μm, 150 Å)) was used with a Dionex HPLC system (Ultimate LPG-3000 binary pump and UVD170U Ultraviolet detector). Modules were controlled by Chromeleon version 6.7. Solvent A (98% water, 2% Acetonitrile) and solvent B (90% Acetonitrile and 10% water) were adjusted to pH 10 using ammonium hydroxide. Samples were injected manually through a Rheodyne valve onto the RP-HPLC column equilibrated with 4% solvent B and kept at this percentage for 6 minutes. A two step gradient was applied at a flow-rate of 1 ml/min (from 4–28% B in 36 minutes, then from 28-50% B in 8 minutes) followed by a 5-minute washing step at 80% solvent B and a 10-minute re-equilibration step, for a total run time of 65 minutes. Column eluate was monitored at 220 and 280 nm, and collected using a Foxy Jr. FC144 fraction collector (Dionex). Collection was allowed from 8 to 50 minutes for 85 seconds per vial (1.42 ml) for a total of 30 fractions. No fraction concatenation strategy was used; only the first 4 and the last 5 fractions were pooled resulting in 21 fractions in total. Previous analysis had shown that 21 fractions provided the highest number of peptide identifications after analysis using the MaxQuant and Perseus pipeline.

#### Proteomics LC-MS protocol

Each of the 21 fractions was then re-suspended in 2% acetonitrile/0.1% TFA acid in water and separated by nanoscale C18 reverse-phase liquid chromatography performed on an EASY-nLC II (Thermo Scientific) coupled to a Linear Trap Quadrupole - Orbitrap Velos mass spectrometer (Thermo Scientific). Elution was carried out using a binary gradient with buffer A: 2% acetonitrile and B: 80% acetonitrile, both containing 0.1% of formic acid. Peptides were subsequently eluted at 200 nl/min flow, into a 20 cm fused silica emitter (New Objective) packed in-house with ReproSil-Pur C18-AQ, 1.9 μm resin (Dr Maisch GmbH). Packed emitter was kept at 35°C by means of a column oven integrated into the nanoelectrospray ion source (Sonation). Peptides were eluted at a flow rate of 200 nl/min using 3 different gradients optimised for set of fractions 1-7 (2,20,41% buffer B), 8-15 (5, 25, 46% buffer B) and 16-21 (7, 28, 50% buffer B). Two-step gradients were used, all with 42 minutes for step one and 13 minutes for step two.All gradients were followed by a washing step (100% B) for 10 minutes followed by a 20 minute re-equilibration step (5%), for a total run time of 85 minutes.

Eluting peptides were electrosprayed into the mass spectrometer using a nanoelectrospray ion source (Thermo Scientific). An Active Background Ion Reduction Device was used to decrease air contaminants signal level.

#### Proteomics data acquisition

General mass spectrometric conditions were as follows: spray voltage, 2.4 kV, ion transfer tube temperature, 200°C. The mass spectrometer was operated in positive ion mode and used in data-dependent acquisition mode (DDA). A full scan (FT-MS) was acquired at a target value of 1,000,000 ions with resolution R = 60,000 over mass range of 350-1600 amu. The top ten most intense ions were selected for fragmentation in the linear ion trap using Collision Induced Dissociation (CID) using a maximum injection time of 25 ms or a target value of 4000 ions. Multiply charged ions from two to five charges having intensity greater than 3000 counts were selected trough a 1 amu window and fragmented using normalized collision energy of 36 for 10 ms. Former target ions selected for MS/MS were dynamically excluded for 60 seconds.

#### MaxQuant and perseus analysis

Raw data obtained (378 Raw data files in total) were processed with MaxQuant version 1.5.6.3 and searched with Andromeda search engine, querying either Uniprot *Homo sapiens* (UP000000589) and *Mus musculus* (UP000005640) alone or with additional custom FASTA generated from RNA sequencing of the same tumours.

Protein hits coming from individual database were separated in MaxQuant. The “Re-quantify” and “Match Between Runs” options were also used. For quantification, multiplicity was set to 2 (doublets) and Arg0/Arg10, Lys0/Lys8 were used for ratio calculation of SILAC labelled peptides. Only unique peptides were used for protein group quantification. Digestion mode was set to trypsin and allowing for two miscleavages. Iodoacetamide derivative of cysteine were specified as a fixed modification, whereas: oxidation of methionine and acetylation of proteins N-terminus were specified as variable modifications. First and main searches were carried out with precursor mass tolerances of 20 and 4.5 ppm respectively, and the MS/MS tolerance was set to 0.5 Da for CID data. The peptide, protein and site FDR were 0.01; peptides with less than seven amino acid residues were excluded from processing.

Only protein groups identified with at least one unique peptide were used for quantification. The protein groups output file was then loaded into the Perseus platform version 1.6.2.3. Perseus was used to filter the data for confident identifications based on at least 1 unique peptide match and identified in at least 2 of 3 biological replicates in at least one group. A further median normalisation was performed on all samples prior to Welch’s t-test with permutation based FDR set at 0.01 used to identify significantly changing proteins. A further median normalisation of all samples to zero was employed prior to applying FDR (0.01) adjusted statistical testing using Welch’s T-test between HN and CRPC samples. Data were then exported into R version 3.5.2 for downstream analysis.

#### Reactome pathways GSEA

Data were exported into R and the following packages used for downstream analysis, VennDiagram, splitstackshape, biomaRt, fgsea, ggplot2, FGNet, stringr, dplyr. Column means of normalised Light / Heavy SILAC ratio were generated as a measure of the increase in abundance of protein in the CRPC model compared to its HN tumour. In summary ratio change between HN and CRPC models were used to generate ranked lists for GSEA (FGSEA package) analysis using Reactome pathways.

#### RNA sequencing

RNA samples were depleted for both cytoplasmic and mitochondrial RNA using the Ribo-Zero gold illumina kit and sequenced on an Illumina NextSeq 500 using High Output 75 cycles kit (2 × 36 cycles, paired end reads, single index). FastQ files were generated using Illumina’s bcl2fastq (v. 2.20.0.422), read quality was checked with FastQC (v. 0.11.7) outputs available via Mendeley data portal. Alignment to the GRCh38 human genome was performed with Tophat (v. 2.1.0). Differential expression with normalisation based on negative binomial distribution was performed in DESeq2 prior to downstream analysis in R.

### Quantification and statistical analysis

#### Gene set enrichment and multi-omics analysis

21,499 transcripts and 4,470 proteins were converted to ranked gene lists by normalised Log_2_ SILAC ratio change or normalised Log_2_ FC reads in CRPC for input into the Reactome GSEA analysis (R package clusterProfiler). Annotated compounds via Compound Discoverer (MZ Cloud) were mapped onto Reactome pathways of interest and identification manually confirmed by mass, RT and fragmentation spectra (Data S4). Heatmaps were generated in R using package pheatmap. Welch’s t-tests were used with Benjamini Hochberge or permutation based FDR p-value adjustment for multiple comparisons.

## Data Availability

The datasets and code generated for this study are available at publicly available repositories. Proteomics data via ProteomeXchange (PXD021428) and Mendeley data. RNA sequencing data via ArrayExpress (E-MTAB-9831) and Mendeley data. Metabolomics data via Mendeley data.

## Supplemental information

Document S1. Figure S1

Data S1. Details of 112 compounds identified by targeted metabolomics analysis, along with additional validation analysis using standard for NAA and NAAG (second sheet)

Data S2. Full names of pathways not shown in full names in Figure 3C and 3D

Data S3. Summary of patient-related parameters in the clinical cohorts included in Figure 4C

Data S4. List of manually confirmed compounds following Compound Discoverer analysis

## References

[bib1] Baslow M.H. (2002). Functions of N-Acetyl-l-Aspartate and N-Acetyl-l-Aspartylglutamate in the vertebrate brain. J. Neurochem..

[bib2] Berthois Y., Katzenellenbogen J.A., Katzenellenbogen B.S. (1986). Phenol red in tissue culture media is a weak estrogen: implications concerning the study of estrogen-responsive cells in culture. Proc. Natl. Acad. Sci. U S A.

[bib3] Blomme A., Ford C.A., Mui E., Patel R., Ntala C., Jamieson L.E., Planque M., McGregor G.H., Peixoto P., Hervouet E. (2020). 2,4-dienoyl-CoA reductase regulates lipid homeostasis in treatment-resistant prostate cancer. Nat. Commun..

[bib4] Boersema P.J., Geiger T., Wisniewski J.R., Mann M. (2013). Quantification of the N-glycosylated secretome by super-SILAC during breast cancer progression and in human blood samples. Mol. Cell. Proteomics.

[bib5] Bogner-Strauss J.G. (2017). N-acetylaspartate metabolism outside the brain: lipogenesis, histone acetylation, and cancer. Front. Endocrinol..

[bib6] Davies L.C., Rice C.M., Palmieri E.M., Taylor P.R., Kuhns D.B., McVicar D.W. (2017). Peritoneal tissue-resident macrophages are metabolically poised to engage microbes using tissue-niche fuels. Nat. Commun..

[bib7] Edden R.A.E., Pomper M.G., Barker P.B. (2007). In vivo differentiation of N-acetyl aspartyl glutamate from N-acetyl aspartate at 3 Tesla. Magn. Reson. Med..

[bib8] Geng H., Xue C., Mendonca J., Sun X.-X., Liu Q., Reardon P.N., Chen Y., Qian K., Hua V., Chen A. (2018). Interplay between hypoxia and androgen controls a metabolic switch conferring resistance to androgen/AR-targeted therapy. Nat. Commun..

[bib9] Grasso C.S., Wu Y.-M., Robinson D.R., Cao X., Dhanasekaran S.M., Khan A.P., Quist M.J., Jing X., Lonigro R.J., Brenner J.C. (2012). The mutational landscape of lethal castration-resistant prostate cancer. Nature.

[bib10] Gucalp A., Iyengar N.M., Zhou X.K., Giri D.D., Falcone D.J., Wang H., Williams S., Krasne M.D., Yaghnam I., Kunzel B. (2017). Periprostatic adipose inflammation is associated with high-grade prostate cancer. Prostate Cancer Prostatic Dis..

[bib11] Hieronymus H., Schultz N., Gopalan A., Carver B.S., Chang M.T., Xiao Y., Heguy A., Huberman K., Bernstein M., Assel M. (2014). Copy number alteration burden predicts prostate cancer relapse. Proc. Natl. Acad. Sci. U S A.

[bib12] Horoszewicz J.S., Leong S.S., Chu T.M., Wajsman Z.L., Friedman M., Papsidero L., Kim U., Chai L.S., Kakati S., Arya S.K., Sandberg A.A. (1980). The LNCaP cell line--a new model for studies on human prostatic carcinoma. Prog. Clin. Biol. Res..

[bib13] Huber K., Hofer D.C., Trefely S., Pelzmann H.J., Madreiter-Sokolowski C., Duta-Mare M., Schlager S., Trausinger G., Stryeck S., Graier W.F. (2019). N-acetylaspartate pathway is nutrient responsive and coordinates lipid and energy metabolism in brown adipocytes. Biochim. Biophys. Acta Mol. Cell Res..

[bib14] Kim D., Pertea G., Trapnell C., Pimentel H., Kelley R., Salzberg S.L. (2013). TopHat2: accurate alignment of transcriptomes in the presence of insertions, deletions and gene fusions. Genome Biol..

[bib15] Komatsu S., Hara N., Ishizaki F., Nishiyama T., Takizawa I., Isahaya E., Kawasaki T., Takahashi K. (2012). Altered association of interleukin-6 with sex steroids in lipid metabolism disorder in men with prostate cancer receiving androgen deprivation therapy. Prostate.

[bib16] Korenchuk S., Lehr J.E., MClean L., Lee Y.G., Whitney S., Vessella R., Lin D.L., Pienta K.J. (2001). VCaP, a cell-based model system of human prostate cancer. In Vivo.

[bib17] Kuntz E.M., Baquero P., Michie A.M., Dunn K., Tardito S., Holyoake T.L., Helgason G.V., Gottlieb E. (2017). Targeting mitochondrial oxidative phosphorylation eradicates therapy-resistant chronic myeloid leukemia stem cells. Nat. Med..

[bib18] Liu Y., Beyer A., Aebersold R. (2016). On the dependency of cellular protein levels on mRNA abundance. Cell.

[bib19] Love M.I., Huber W., Anders S. (2014). Moderated estimation of fold change and dispersion for RNA-seq data with DESeq2. Genome Biol..

[bib20] Martinez R.S., Salji M.J., Rushworth L., Ntala C., Rodriguez Blanco G., Hedley A., Clark W., Peixoto P., Hervouet E., Renaude E. (2020). Schlafen family member 5 (SLFN5) regulates LAT1-mediated mTOR activation in castration-resistant prostate cancer. BioRxiv.

[bib21] Mehta V., Namboodiri M.A. (1995). N-acetylaspartate as an acetyl source in the nervous system. Brain Res. Mol. Brain Res..

[bib22] Meller B., Bremmer F., Sahlmann C.O., Hijazi S., Bouter C., Trojan L., Meller J., Thelen P. (2015). Alterations in androgen deprivation enhanced prostate-specific membrane antigen (PSMA) expression in prostate cancer cells as a target for diagnostics and therapy. EJNMMI Res..

[bib23] Meller S., Meyer H.-A., Bethan B., Dietrich D., Maldonado S.G., Lein M., Montani M., Reszka R., Schatz P., Peter E. (2016). Integration of tissue metabolomics, transcriptomics and immunohistochemistry reveals ERG- and gleason score-specific metabolomic alterations in prostate cancer. Oncotarget.

[bib24] Moffett J., Tieman S.B., Weinberger D.R., Coyle J.T., Namboodiri A.M.A. (2006).

[bib25] Nguyen T., Kirsch B.J., Asaka R., Nabi K., Quinones A., Tan J., Antonio M.J., Camelo F., Li T., Nguyen S. (2019). Uncovering the role of N-Acetyl-Aspartyl-Glutamate as a glutamate reservoir in cancer. Cell Rep..

[bib26] Oakes S.A., Papa F.R. (2015). The role of endoplasmic reticulum stress in human pathology. Annu. Rev. Pathol..

[bib27] Patel R., Fleming J., Mui E., Loveridge C., Repiscak P., Blomme A., Harle V., Salji M., Ahmad I., Teo K. (2018). Sprouty2 loss-induced IL6 drives castration-resistant prostate cancer through scavenger receptor B1. EMBO Mol. Med..

[bib28] Ribeiro R., Monteiro C., Cunha V., Oliveira M.J., Freitas M., Fraga A., Príncipe P., Lobato C., Lobo F., Morais A. (2012). Human periprostatic adipose tissue promotes prostate cancer aggressiveness in vitro. J. Exp. Clin. Cancer Res..

[bib29] Sciarra A., Gentilucci A., Salciccia S., Pierella F., Del Bianco F., Gentile V., Silvestri I., Cattarino S. (2016). Prognostic value of inflammation in prostate cancer progression and response to therapeutic: a critical review. J. Inflamm..

[bib30] Sharifi N. (2013). Minireview: androgen metabolism in castration-resistant prostate cancer. Mol. Endocrinol..

[bib31] Simpson B.S., Camacho N., Luxton H.J., Pye H., Finn R., Heavey S., Pitt J., Moore C.M., Whitaker H.C. (2020). Genetic alterations in the 3q26.31-32 locus confer an aggressive prostate cancer phenotype. Commun. Biol..

[bib32] Sircar K., Huang H., Hu L., Cogdell D., Dhillon J., Tzelepi V., Efstathiou E., Koumakpayi I.H., Saad F., Luo D. (2012). Integrative molecular profiling reveals asparagine synthetase is a target in castration-resistant prostate cancer. Am. J. Pathol..

[bib33] Smittenaar C.R., Petersen K.A., Stewart K., Moitt N. (2016). Cancer incidence and mortality projections in the UK until 2035. Br. J. Cancer.

[bib34] Sramkoski R.M., Pretlow T.G., Giaconia J.M., Pretlow T.P., Schwartz S., Sy M.S., Marengo S.R., Rhim J.S., Zhang D., Jacobberger J.W. (1999). A new human prostate carcinoma cell line, 22Rv1. In Vitro Cell Dev. Biol. Anim..

[bib35] Sreekumar A., Poisson L.M., Rajendiran T.M., Khan A.P., Cao Q., Yu J., Laxman B., Mehra R., Lonigro R.J., Li Y. (2009). Metabolomic profiles delineate potential role for sarcosine in prostate cancer progression. Nature.

[bib36] Stagljar I. (2016). The power of OMICs. Biochem. Biophys. Res. Commun..

[bib37] Taylor B.S., Schultz N., Hieronymus H., Gopalan A., Xiao Y., Carver B.S., Arora V.K., Kaushik P., Cerami E., Reva B. (2010). Integrative genomic profiling of human prostate cancer. Cancer Cell.

[bib38] Taylor R.A., Lo J., Ascui N., Watt M.J. (2015). Linking obesogenic dysregulation to prostate cancer progression. Endocr. Connect..

[bib39] Tyanova S., Temu T., Cox J. (2016). The MaxQuant computational platform for mass spectrometry-based shotgun proteomics. Nat. Protoc..

[bib40] Tyanova S., Temu T., Sinitcyn P., Carlson A., Hein M.Y., Geiger T., Mann M., Cox J. (2016). The Perseus computational platform for comprehensive analysis of (prote)omics data. Nat. Methods.

[bib41] Voelkel-Johnson C., Norris J.S., White-Gilbertson S. (2018). Interdiction of sphingolipid metabolism revisited: focus on prostate cancer. Adv. Cancer Res..

[bib42] Wang Y., Yang F., Gritsenko M.A., Wang Y., Clauss T., Liu T., Shen Y., Monroe M.E., Lopez-Ferrer D., Reno T. (2011). Reversed-phase chromatography with multiple fraction concatenation strategy for proteome profiling of human MCF10A cells. Proteomics.

[bib43] Whitaker H.C., Shiong L.L., Kay J.D., Grönberg H., Warren A.Y., Seipel A., Wiklund F., Thomas B., Wiklund P., Miller J.L. (2014). N-acetyl-L-aspartyl-L-glutamate peptidase-like 2 is overexpressed in cancer and promotes a pro-migratory and pro-metastatic phenotype. Oncogene.

[bib44] Wiame E., Tyteca D., Pierrot N., Collard F., Amyere M., Noel G., Desmedt J., Nassogne M.-C., Vikkula M., Octave J.-N. (2009). Molecular identification of aspartate N-acetyltransferase and its mutation in hypoacetylaspartia. Biochem. J..

