# Supplementary material for: Multi-omics & pathway analysis identify potential roles for tumor N-acetyl aspartate accumulation in murine models of castration-resistant prostate cancer
Source: iScience. 2022 Mar 11;25(4):104056. doi: 10.1016/j.isci.2022.104056 (PMC8957019; doi:10.1016/j.isci.2022.104056)
Supplement: Document S1. Figure S1 [file mmc1.pdf]

iScience, Volume *25*

Supplemental information

Multi-omics & pathway analysis identify potential roles for tumor N-acetyl aspartate accumulation in murine models of castration-resistant prostate cancer

Mark J. Salji, Arnaud Blomme, J. Henry M. Däbritz, Peter Repiscak, Sergio Lilla, Rachana Patel, David Sumpton, Niels J.F. van den Broek, Ronan Daly, Sara Zanivan, and Hing Y. Leung

Supplementary Figure S1. Untargeted Metabolomics of Orthotopic Tumour Models. Related to Figure 1.

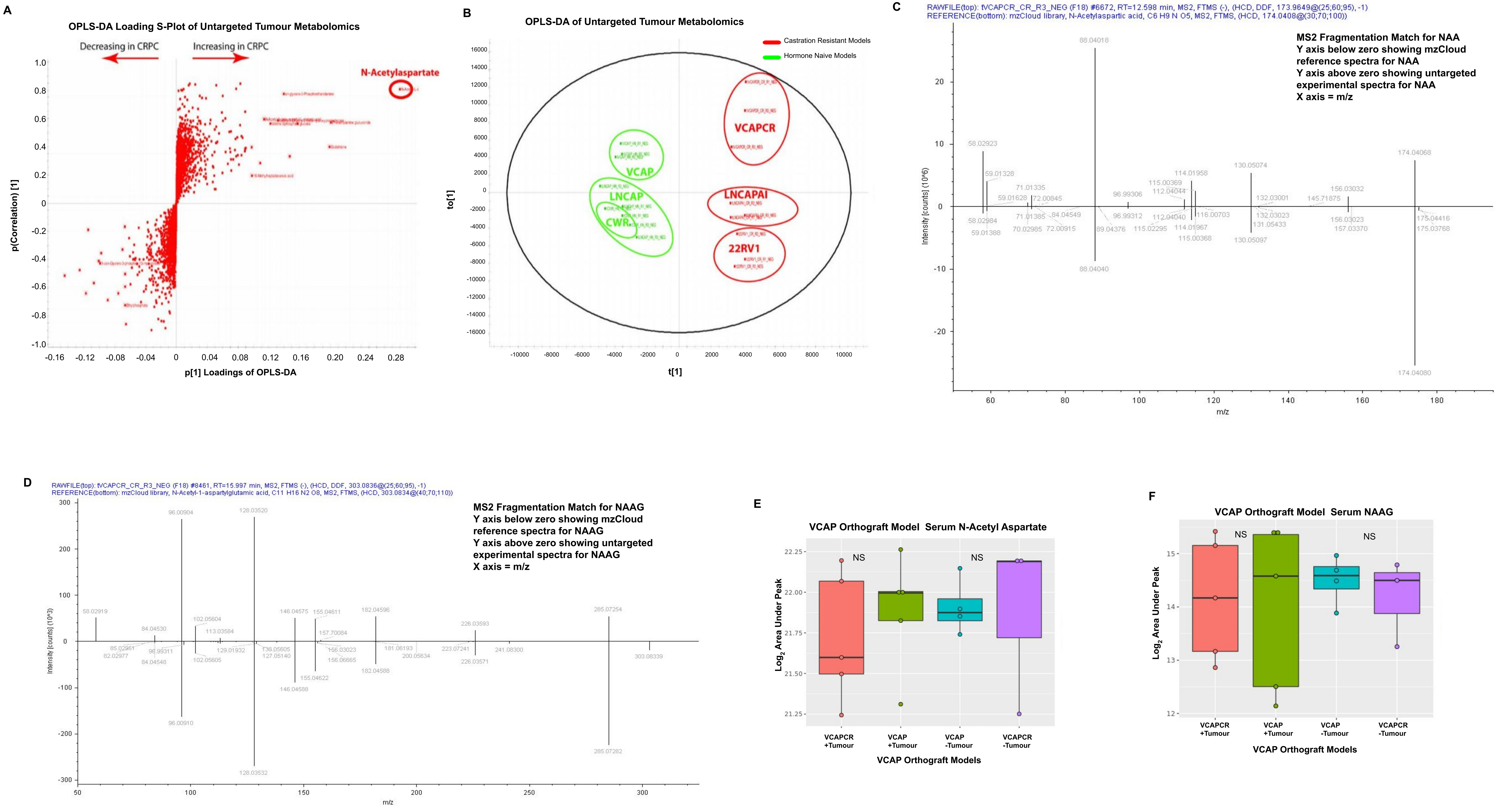

Legend to Supplementary Figure S1

A. OPLSDA loading S-plot of untargeted metabolomics Progenesis analysis of negative ion mode. NAA position is shown in top right.
B. OPLSDA scatter plot of untargeted metabolomics Progenesis analysis of negative ion mode. Clustering of the PC models and separation of HN and CRPC models is shown by the OPLSDA.
C. MS2 fragmentation spectra match of NAA by untargeted metabolomics using Compound Discoverer analysis of negative ion mode. Reference NAA spectra from mzCloud is shown above zero and experimental MS2 fragments is shown below zero.
D. MS2 fragmentation spectra match of NAAG by untargeted metabolomics using Compound Discoverer analysis of negative ion mode. Reference NAAG spectra from mzCloud is shown above zero and experimental MS2 fragments is shown below zero.
E. Targeted metabolomics of serum from VCAP and VCAPCR tumour bearing CD-1 nude orthotopic models. NAA and NAAG levels are unchanged in the serum between HN and CRPC tumour bearing models, in contrast to NAA and NAAG being increased in CRPC tumours (Figure 1). NAA and NAAG levels are also unchanged in the serum between tumour bearing and non-tumour bearing mice (serum from failed orthotopic injections). Box and whisker plots midline represents the median Log_2 area under peak (AUC) with hinges representing the 1st and 3rd quartiles and whiskers extending to 1.5x the IQR with all data points (biological replicates) including outliers shown.
